# Longer-Term Outcomes Following Mechanical Thrombectomy for Intermediate- and High-Risk Pulmonary Embolism: 6-Month FLASH Registry Results

**DOI:** 10.1016/j.jscai.2023.101000

**Published:** 2023-05-19

**Authors:** Sameer Khandhar, Wissam Jaber, Matthew C. Bunte, Kenneth Cho, Mitchell D. Weinberg, Bushra Mina, Brian Stegman, Jeffrey Pollak, Akhil Khosla, Fakhir Elmasri, David Zlotnick, Daniel Brancheau, Gerald Koenig, Mohannad Bisharat, Jun Li, Catalin Toma

**Affiliations:** a Division of Cardiovascular Medicine, Penn Presbyterian Medical Center, Perelman School of Medicine at the University of Pennsylvania, Philadelphia, Pennsylvania; b Emory University Hospital, Atlanta, Georgia; c Saint Luke’s Mid America Heart Institute, Kansas City, Missouri; d Department of Cardiology, Northwell Health, Zucker School of Medicine at Hofstra/Northwell, Staten Island University Hospital, Staten Island, New York; e Department of Pulmonary Critical Care Medicine, Lenox Hill Hospital, Northwell Health, Zucker School of Medicine at Hofstra/Northwell, New York, New York; f CentraCare Heart and Vascular Center, St Cloud, Minnesota; g Department of Radiology and Biomedical Imaging, Yale University, New Haven, Connecticut; h Department of Pulmonary, Critical Care and Sleep Medicine, Yale University, Yale New Haven Hospital, New Haven, Connecticut; i Division of Interventional Radiology, Lakeland Regional Medical Center, Lakeland, Florida; j Division of Cardiovascular Medicine, University at Buffalo, Gates Vascular Institute, Buffalo General Medical Center, Buffalo, New York; k Ascension Genesys Hospital, Grand Blanc, Michigan; l Division of Cardiovascular Medicine, Henry Ford Health System, Wayne State University School of Medicine, Detroit, Michigan; m Memorial Hospital Jacksonville, Jacksonville, Florida; n Harrington Heart and Vascular Institute, University Hospitals, Cleveland, Ohio; o Heart and Vascular Institute, University of Pittsburgh Medical Center, Pittsburgh, Pennsylvania

**Keywords:** mechanical thrombectomy, long-term outcomes, percutaneous intervention, pulmonary embolism

## Abstract

**Background:**

Mechanical thrombectomy provides rapid hemodynamic improvements after acute pulmonary embolism (PE), but long-term benefits are uncertain.

**Methods:**

FlowTriever All-comer Registry for Patient Safety and Hemodynamics is a prospective, single-arm, multicenter registry of patients with acute PE treated with the FlowTriever System (Inari Medical). Six-month outcomes including modified Medical Research Council dyspnea scores (MMRCD), right ventricular (RV) function, 6-minute walk test distances, and PE quality-of-life scores (QoL) were assessed.

**Results:**

In total, 799 patients were enrolled and 75% completed the study with a mean follow-up of 204 ± 46 days. Demographic characteristics included 54.1% men, mean age of 61.2 years, 77.1% intermediate-high-risk PE, and 8.0% high-risk PE. All-cause mortality was 4.6% at study completion. The proportion of patients with normal echocardiographic RV function increased from 15.1% at baseline to 95.1% at 6 months (*P* < .0001). MMRCD score improved from 3.0 at baseline to 0.0 at 6 months (*P* < .0001). 6-minute walk test distances increased from 180 m at 48 hours to 398 m at 6 months (*P* < .001). Median PE QoL total scores were 9.38 at 30 days and 4.85 at 6 months (*P* < .001). Prevalence of site-reported chronic thromboembolic pulmonary hypertension was 1.0% and chronic thromboembolic disease was 1.9%.

**Conclusions:**

In this large diverse group of PE patients, 6-month all-cause mortality, chronic thromboembolic pulmonary hypertension, and chronic thromboembolic disease were low following thrombectomy with the FlowTriever system. Significant improvements in RV function, patient symptoms, exercise capacity, and QoL were observed at 6 months, suggesting that rapid extraction of thrombus may prevent long-term sequelae in patients with PE.

## Highlights

• FlowTriever All-comer Registry for Patient Safety and Hemodynamics pulmonary embolism outcomes are reported at 6 months postthrombectomy.
• All-cause mortality was 4.6% (29/628) and long-term pulmonary embolism sequelae were infrequent.
• At the 6-month visit, multiple clinical and functional improvements were reported.
• 95.1% had normal right ventricular function on echo and 90.1% had no or only mild dyspnea symptoms.
• Median 6-minute walk test distance was 398 m and median total Pulmonary Embolism quality-of-life score was 4.85.
• Improvements were predominantly achieved within 30 days and maintained to 6 months.

## Central Illustration

**Figure:**
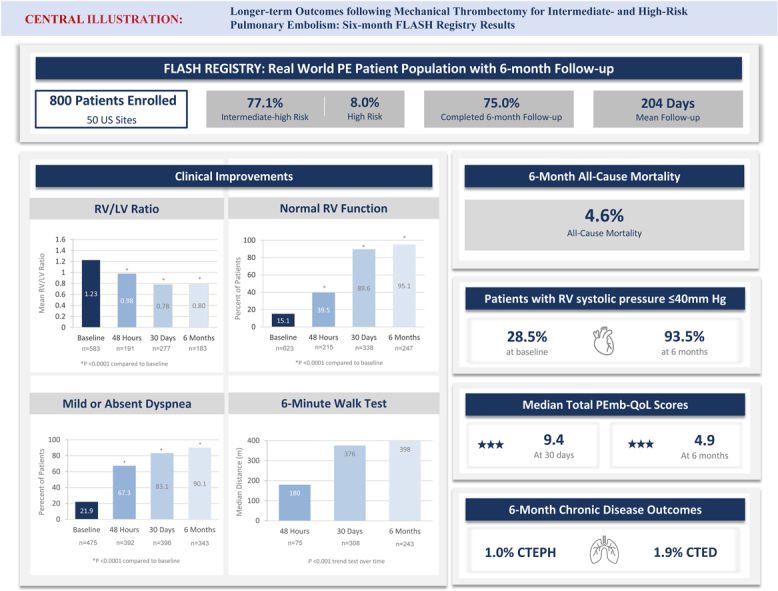

## Introduction

Pulmonary embolism (PE) remains a clinically challenging disease that is associated with both short- and long-term morbidity and mortality. Given the acuity of PE, studies evaluating interventional treatments have focused on establishing short-term safety, improving short-term right ventricular (RV) hemodynamics, preventing hemodynamic decompensation, and reducing in-hospital death. However, survivors of PE can have significant limitations late after diagnosis, including persistent dyspnea, impaired exercise capacity, and reduced quality of life (QoL),^1^ known as post-PE impairment (PPEI). This long-term functional pulmonary impairment can impact up to 50% of PE survivors^1^ and encompasses a spectrum of diseases with increasing severity and varying presentations ranging from post-PE syndrome or chronic thromboembolic disease (CTED) to chronic thromboembolic pulmonary hypertension (CTEPH).^2^ CTED is characterized by persistent dyspnea in the absence of resting pulmonary hypertension and may be associated with residual pulmonary vascular obstruction (RPVO). CTEPH, the most severe form of PPEI, is manifested by persistent dyspnea, RPVO, and resting pulmonary hypertension, all of which are associated with a high rate of morbidity and mortality.^3^ Both CTED and CTEPH are associated with a significant reduction in QoL and portend a worse prognosis.

Over the past decade, catheter-based treatment options for acute PE have evolved beyond traditional thrombolytic treatments to include large-bore mechanical thrombectomy to disrupt and aspirate PE thrombus. The FlowTriever System (Inari Medical) has demonstrated favorable acute safety and effectiveness for treating PE, including acutely reduced pulmonary artery pressure (PAP), improved RV function, and increased cardiac output during the index hospitalization.^4^, ^5^, ^6^, ^7^, ^8^, ^9^ However, the longer-term benefits of large-bore mechanical thrombectomy are less certain.

The FlowTriever All-Comer Registry for Patient Safety and Hemodynamics (FLASH) is a prospective, multicenter, single-arm study to evaluate the acute and longer-term safety and effectiveness of the FlowTriever System among a large and geographically diverse population with acute PE. In-hospital and 30-day outcomes from the fully enrolled US cohort of 800 patients have been published previously.^10^ This report focuses on the longer-term outcomes, including late all-cause mortality, RV remodeling, residual symptoms, functional status, and PE-specific QoL through 6-month follow-up after treatment with the FlowTriever System.

## Methods

### Study design

FLASH is an all-comer, prospective, multicenter registry (ClinicalTrials.gov identifier: NCT03761173) to evaluate real-world outcomes in patients with PE treated with mechanical thrombectomy using the FlowTriever System. The FlowTriever System is composed of 2 main components: the Triever aspiration catheter used for controlled aspiration of thromboemboli, and the FlowTriever catheter comprised of self-expanding nitinol disks designed to engage the thrombus for removal via aspiration.

Details of the FLASH registry study design have been previously reported.^9^^,^^10^ Inclusion criteria were limited to patients aged ≥18 years old with acute intermediate- or high-risk PE per European Society of Cardiology guidelines.^11^ Exclusion criteria included patients unable to be anticoagulated and life expectancy of <30 days. Investigators obtained institutional review board approval at each site prior to enrolling patients, and all patients provided written informed consent. Follow-up assessments occurred at 48 hours, 30 days, and 6 months postthrombectomy. When patients were unable to attend an in-person follow-up assessment because of COVID-19 or other restrictions, every effort was made to perform a telehealth appointment to measure those parameters suitable to this type of assessment. All safety events and their relatedness to the study device and/or procedure were adjudicated by a third-party independent medical monitor, including all serious adverse events (SAEs) occurring throughout the study follow-up period. SAEs were events meeting the definition of serious in ISO14155,^12^ which included any event that resulted in death, was life-threatening, resulted in or prolonged hospitalization, resulted in significant disability/incapacity or permanent impairment of a bodily function, or necessitated medical or surgical intervention.

### Clinical outcomes assessed

Follow-up visits included echocardiography examinations to evaluate recovery of RV function, dyspnea symptom assessment, exercise capacity evaluation via 6-minute walk tests (6MWT), QoL measurements, and adverse event screening. Echocardiography examinations, 6MWT, and QoL measurements were required at 30 days and 6 months but were optional at 48 hours. Evaluation for dyspnea symptoms and adverse event assessments were required at every follow-up visit.

Echocardiographic examinations included evaluation of RV systolic pressure, size, and function, and RV/left ventricular (RV/LV) ratio, which were evaluated at the site level by physicians using the site’s standard clinical practice. RV function was scored as either normal or mildly, moderately, or severely reduced. Dyspnea assessment used the modified Medical Research Council scale, a self-assessment from 0 (breathless only on strenuous exercise) to 4 (too breathless to leave house, or breathless when dressing/undressing).^13^ The 6MWTs^14^ were performed to assess exercise capability by recording the total distance walked in meters during a 6-minute interval. The patient was also asked to score their level of dyspnea and fatigue before and after the test using the 10-point Borg Scale.^15^

Patient QoL was assessed using the Pulmonary Embolism Quality of Life (PEmb-QoL) questionnaire.^16^ The questionnaire is a validated, disease-specific assessment that consists of 6 parts, or domains, to assess different aspects of the patients’ lives. Results from each of the individual domains were then incorporated into a total score for each patient,^16^ which was calculated from the mean of all nonmissing domains. Higher scores indicate worse health and outcomes.

Prevalence of CTED and CTEPH at 6 months was also evaluated based on site-reported assessments to determine any longer-term disease sequelae. A diagnosis of CTED required evidence at follow-up of persistent dyspnea but without pulmonary hypertension (mean PAP <25 mm Hg). A diagnosis of CTEPH required evidence at follow-up of persistent dyspnea, pulmonary hypertension (mean PAP ≥25 mm Hg), and abnormal imaging, although the study protocol did not specify imaging type.

### Statistical analyses

Data are presented as either proportion (%), mean ± SD, or median (IQR). For variables where baseline measurements were available, Wilcoxon signed-rank test and McNemar’s or McNemar-Bowker’s tests were applied to test the changes from baseline for continuous and categorical outcomes, respectively, using available paired values. For variables where baseline measurements were not available, trend over time analysis was performed using a generalized linear mixed effects model assuming patient-specific random slope and intercept, using all available data. The model was adjusted for the effect of age, body mass index, and sex. Time was computed as the number of days between procedure date and visit date. A 2-sided *P* value of .05 was used to determine statistical significance. Analyses were performed using SAS 9.4 (SAS Institute) and R version 4.1.2.^17^ Kaplan-Meier assessment of freedom from mortality was calculated using R version 4.1.2.^17^

To address the potential effects of missingness on study findings, a sensitivity analysis using mixed models for repeated measures was performed assuming a missing-at-random model. Each outcome of interest incorporated a random intercept of subjects and random slopes of study follow-up visits in addition to relevant covariates. The mean estimates and probability distributions derived from mixed models for repeated measures for each follow-up timepoint had similar trends compared with the actual means and proportion distributions reported in this manuscript. These sensitivity analysis findings are therefore consistent with the missing-at-random assumption.

## Results

### Baseline and procedural characteristics

**Figure 1:**
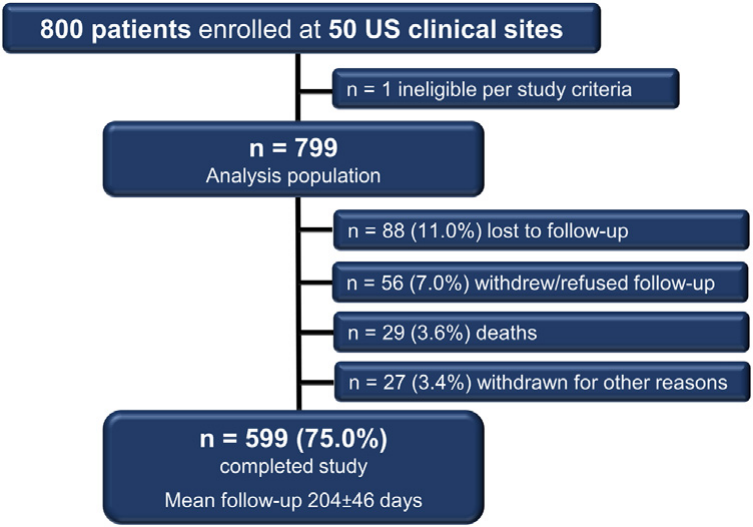
**Flow diagram of patient enrollment and follow-up**.

The FLASH registry enrolled 800 patients treated with the FlowTriever System at 50 sites across the United States between December 2018 and December 2021. The analysis population was 799 patients because 1 patient was deemed ineligible postenrollment for meeting an exclusion criterion (RV/LV ratio of <0.9). A more extensive presentation of baseline and procedural characteristics of this cohort has been previously published.^10^ In brief, 8.0% of the patients had high-risk (massive) PE, and 77.1% had intermediate–high-risk (submassive) PE. Average age was 61.2 ± 14.6 years, and 54.1% of patients were men. The mean simplified Pulmonary Embolism Severity Index was 1.6 ± 1.1, and 32.1% had a relative or absolute contraindication to thrombolytic drugs. Of the 799 patients in the analysis population, 599 (75.0%) completed the study, and the mean follow-up duration of these 599 patients was 204 ± 46 days. Disposition of the remaining patients who did not complete the study is provided in Figure 1.

The immediate improvements in hemodynamics and vital signs, including mean PAP, heart rate, and total pulmonary vascular resistance following thrombectomy have been previously published.^10^ Median (IQR) thrombectomy time was 43 (29-62) minutes. Nineteen patients (2.4%) received adjunctive treatment for PE (18 received catheter-directed thrombolysis [CDT] and 1 received additional percutaneous mechanical thrombectomy).

### Longer-term mortality, safety, and chronic disease outcomes

**Figure 2:**
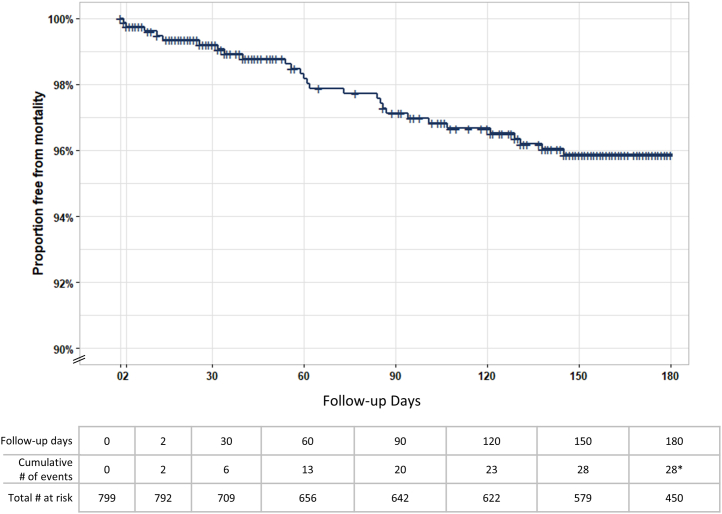
**Kaplan-Meier analysis of freedom from mortality.** Kaplan-Meier time-to-event analysis was performed to evaluate freedom from mortality over time. ∗One additional death occurred at day 291.

There were 2 deaths at 48 hours and 27 additional deaths during the study follow-up, resulting in 4.6% all-cause mortality among the 628 patients with known mortality status at the end of the study (Table 1). Freedom from mortality events determined by Kaplan-Meier analysis is shown in Figure 2. Mortality events occurred at a steady, approximately linear rate throughout the follow-up period, with all deaths occurring prior to 150 days postthrombectomy except for an outlier at 291 days (not shown). A total of 103 SAEs were reported in 84 patients (13.2%) during the study follow-up, 101 of which were adjudicated to be unrelated to the FlowTriever System and the remaining 2 of which were adjudicated to have an unknown relationship to the device. A listing of all SAEs reported throughout the study is provided in Supplemental Table S1. At the 6-month visit, evidence of CTED was reported in 11 patients (1.9%), whereas evidence of CTEPH was reported in 6 patients (1.0%).

**Table 1:** Six-month mortality, safety, and chronic disease outcomes.

Outcome	n/N (%)
All-cause mortality through study exit^a^	29/628 (4.6%)
Patients with SAEs through study exit^b^	84/638 (13.2%)
Prevalence of CTEPH at 6-month visit	6/581 (1.0%)
Prevalence of CTED/post-PE Syndrome at 6-month visit	11/591 (1.9%)

CTED, chronic thromboembolic disease; CTEPH, chronic thromboembolic pulmonary hypertension; PE, pulmonary embolism; SAE, serious adverse event.

a All deaths were adjudicated to be unrelated to the FlowTriever System. The denominator shown represents all patients with known mortality status at the end of the study.

b Two SAEs were adjudicated to have unknown relationship to the FlowTriever System. All other SAEs were unrelated.

### Longer-term anticoagulant use

Among patients completing the 30-day follow-up visit, 89.9% were treated with some form of anticoagulation (AC). This proportion remained similar at the 6-month visit, where 91.2% were receiving AC therapy. The proportions of patients on different types of AC at the 30-day and 6-month follow-up visit are shown in Supplemental Table S2. At the 6-month follow-up visit, 87.2% of patients on AC were being treated with a new oral AC/direct oral AC, with a minority of patients on vitamin K antagonists or other agents.

### RV function outcomes

**Figure 3:**
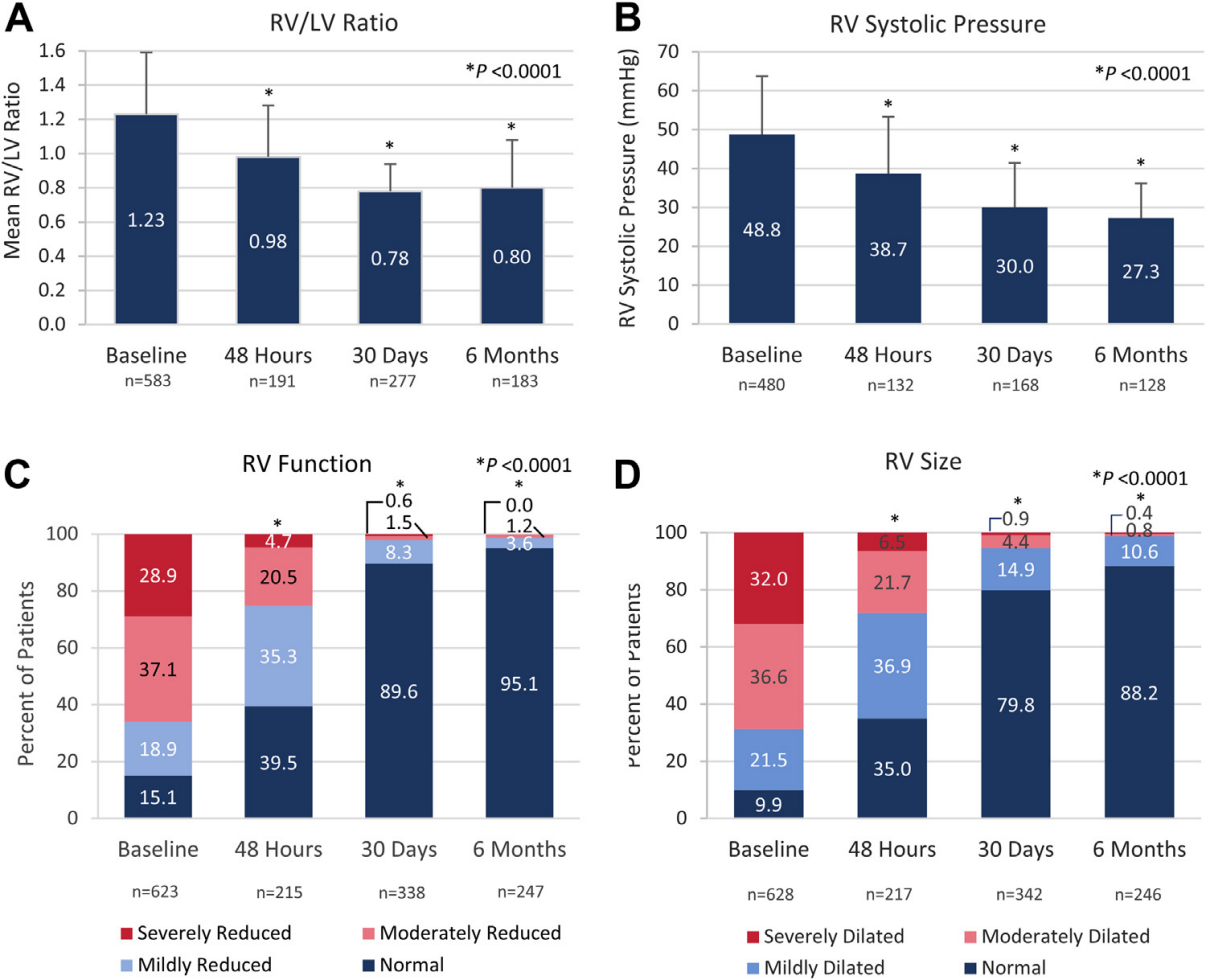
**RV echocardiographic parameters at baseline and 48 hours, 30 days, and 6 months postthrombectomy.** (**A**) Right ventricular (RV) to left ventricular (LV) ratio at baseline and follow-up times (*P* < .0001 for available paired assessments; Wilcoxon signed-rank test). (**B**) RV systolic pressure at baseline and follow-up times (*P* < .0001 for available paired assessments; Wilcoxon signed-rank test). (**C**) Distribution of patients’ RV function at baseline and follow-up times (*P* < .0001 for available paired assessments; McNemar-Bowker’s test). (**D**) Distribution of RV size at baseline and follow-up times (*P* < .0001 for available paired assessments; McNemar-Bowker’s test). The sample sizes below each posttreatment timepoint represent the number of patients who had paired measurements with baseline values.

All prespecified echocardiographic parameters demonstrated significant improvement from baseline to 48 hours, with further improvements continuing out to 6 months (Figure 3). Mean RV/LV ratios improved from 1.23 ± 0.36 at baseline to 0.98 ± 0.30, 0.78 ± 0.16 and 0.80 ± 0.28 at 48 hours, 30 days, and 6 months, respectively (Figure 3A, *P* < .0001). Similarly, mean estimated RV systolic pressure declined from 48.8 ± 14.9 mm Hg at baseline to 38.7 ± 14.6, 30.0 ± 11.4, and 27.3 ± 8.9 mm Hg at 48 hours, 30 days, and 6 months, respectively (Figure 3B, *P* < .0001). The proportion of patients with RV systolic pressure of ≤40 mm Hg improved from 28.5% at baseline to 93.5% at 6 months. The distribution of patients at each RV function level changed significantly over time (Figure 3C, *P* < .0001), with the proportion of patients with normal function increasing from 15.1% at baseline to 95.1% at 6 months. Similarly, RV size also improved significantly over the follow-up period (Figure 3D, *P* < .0001), with the proportion of patients exhibiting normal RV size increasing from 9.9% at baseline to 88.2% at 6 months.

### Dyspnea, QoL, and 6MWT outcomes

**Figure 4:**
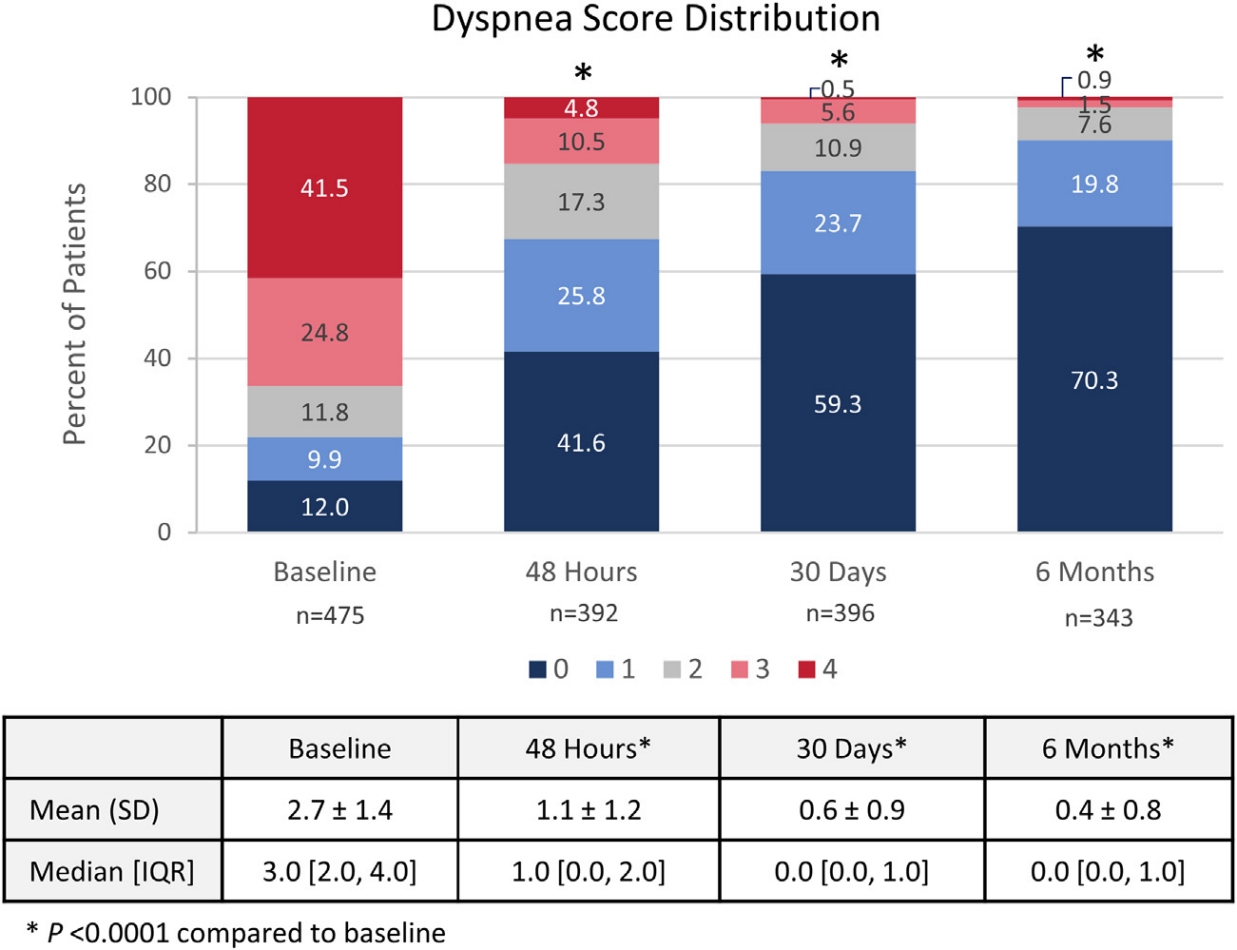
**Dyspnea score distribution at baseline, 48 hours, 30 days, and 6 months postthrombectomy**. Dyspnea was assessed at baseline and at follow-up visits at 48 hours, 30 days, and 6 months postthrombectomy using the modified Medical Research Council assessment tool (higher score = worse dyspnea). The proportion of patients with each score (0-4) is presented, showing a significant change in score distribution (*P* < .0001 for available paired assessments; McNemar-Bowker’s test). Mean ± SD and median (IQR) values for each timepoint are shown in the table below the graph (*P* < .0001 for available paired assessments; Wilcoxon signed-rank test). The sample sizes below each posttreatment timepoint represent the number of patients who had paired measurements with baseline values.

**Figure 5:**
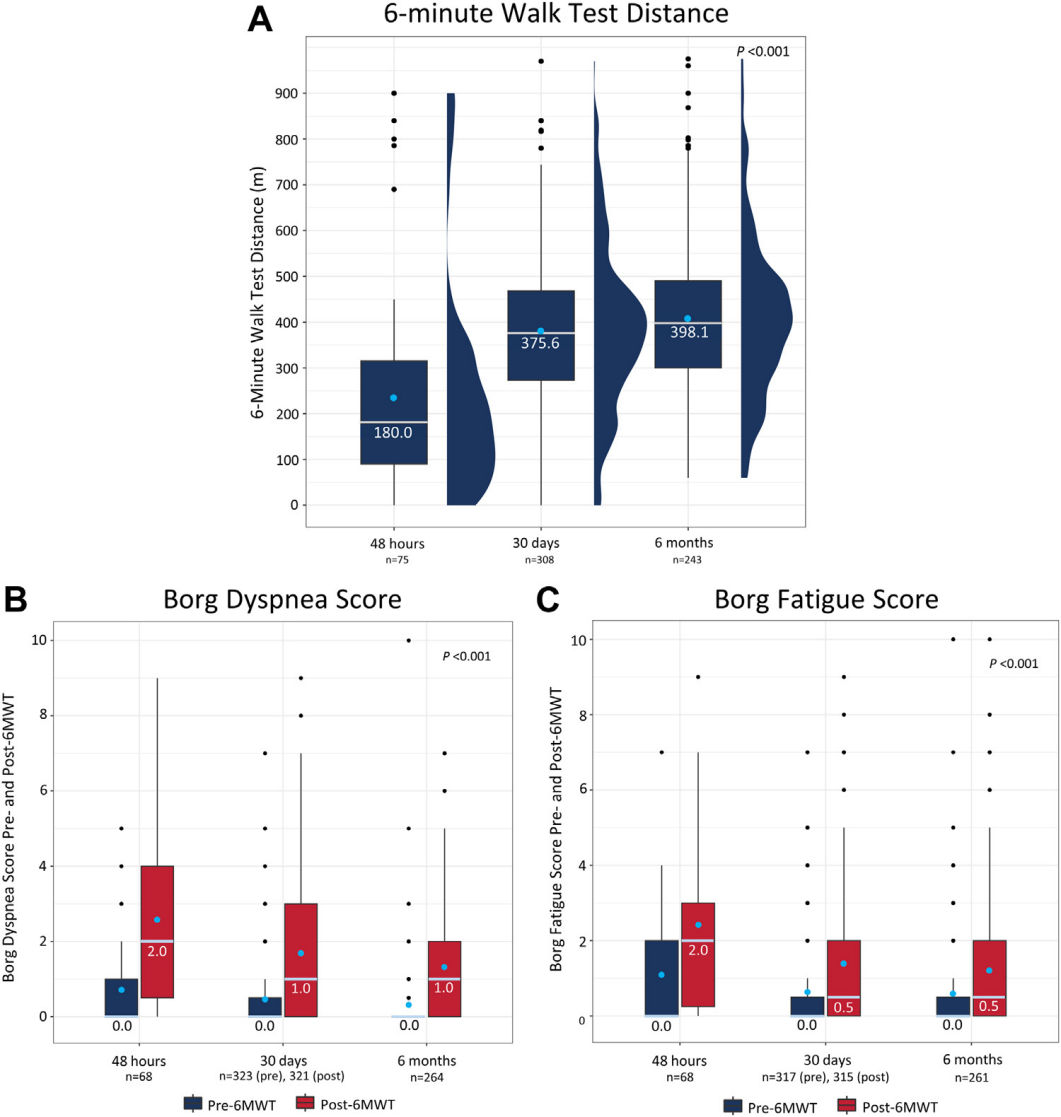
**Six-minute walk test distance and Borg dyspnea and fatigue scores pre- and postwalk at 48 hours, 30 days, and 6 months postthrombectomy.** (**A**) Increase in median 6MWT distance over time. Box and whisker with half-violin plot demonstrates median 6MWT distance in meters (middle bar) and IQR [Q1, Q3] as outer bounds of boxes, with mean indicated as a light blue dot and outliers indicated as black dots. Trend over time analysis was performed using a generalized linear mixed effects model (*P* < .001). (**B**) Borg dyspnea score and (**C**) Borg fatigue score pre- and post-6MWT at follow-up visits. Median scores pre- and post-6MWT at each follow-up visit are shown. Box and whisker plot presents median Borg scores (middle bar) and IQR (Q1-Q3) as outer bounds of boxes, with mean indicated as a light blue dot and outliers indicated as black dots. Trend over time analysis was performed using a generalized linear mixed effects model. Trend over time for dyspnea pre-6MWT: *P* = .004; post-6MWT: *P* < .001. Trend over time for fatigue pre-6MWT: *P* = .064; post-6MWT: *P* < .001. The sample sizes below each timepoint represent all available data. 6MWT, 6-minute walk test.

**Figure 6:**
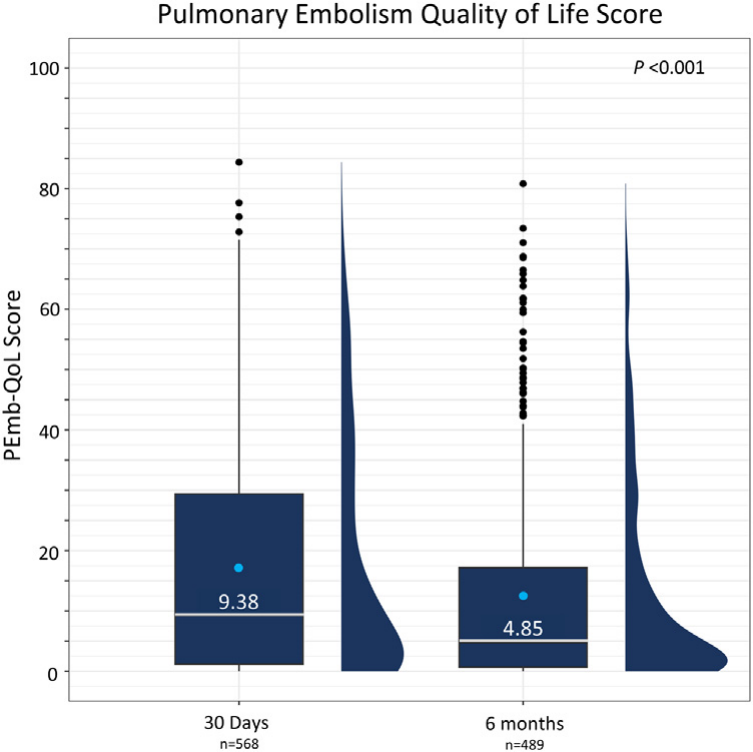
**Total PEmb-QoL score at 30 days and 6 months postthrombectomy.** Box and whisker with half-violin plot demonstrates median PEmb-QoL scores (middle bar) and IQR (Q1-Q3) as outer bounds of boxes, with mean indicated as a light blue dot and outliers indicated as black dots. Trend over time analysis was performed using a generalized linear mixed effects model (*P* < .001). The sample sizes below each timepoint represent all available data. PEmb-QoL, Pulmonary Embolism Quality of Life.

Median (IQR) dyspnea scores decreased from 3.0 (2.0-4.0) at baseline to 1.0 (0.0-2.0) at 48 hours and 0.0 (0.0-1.0) at both 30 days and 6 months. The distribution of dyspnea scores changed significantly over time (Figure 4, *P* < .0001), with the proportion of patients reporting absent or mild dyspnea (score of 0 or 1) increasing from 21.9% at baseline to 67.4% at 48 hours, 83.1% at 30 days, and 90.1% at 6 months. The median values for the 6MWT distances walked at each timepoint are presented in Figure 5A. The median distance walked increased significantly over time from 180.0 (90.0-315.2) m at 48 hours to 375.6)273.0-468.0) m at 30 days, and 398.1 (300.0-490.0) m at 6 months (*P* < .001). Median dyspnea and fatigue scores were 0.0 immediately prior to the 6MWT at 48 hours, which were maintained out to 6 months (Figure 5B, C). Median dyspnea and fatigue scores following the 6MWT were 2.0 at 48 hours, indicating patients had some difficulty with exercise at this early assessment. Notably, the median post-6MWT dyspnea scores were significantly reduced to 1.0 at 30 days and 6 months (*P* < .001), whereas median post-6MWT fatigue scores were reduced to 0.5 at 30 days and 6 months (*P* < .001). Median (IQR) PEmb-QoL total scores were 9.38 (1.22-29.37) at the 30-day visit and 4.85 (0.67-17.18) at the 6-month visit (Figure 6), where lower scores indicated a higher QoL. These key longer-term effectiveness outcomes are summarized in the Central Illustration.

**Central Illustration:**
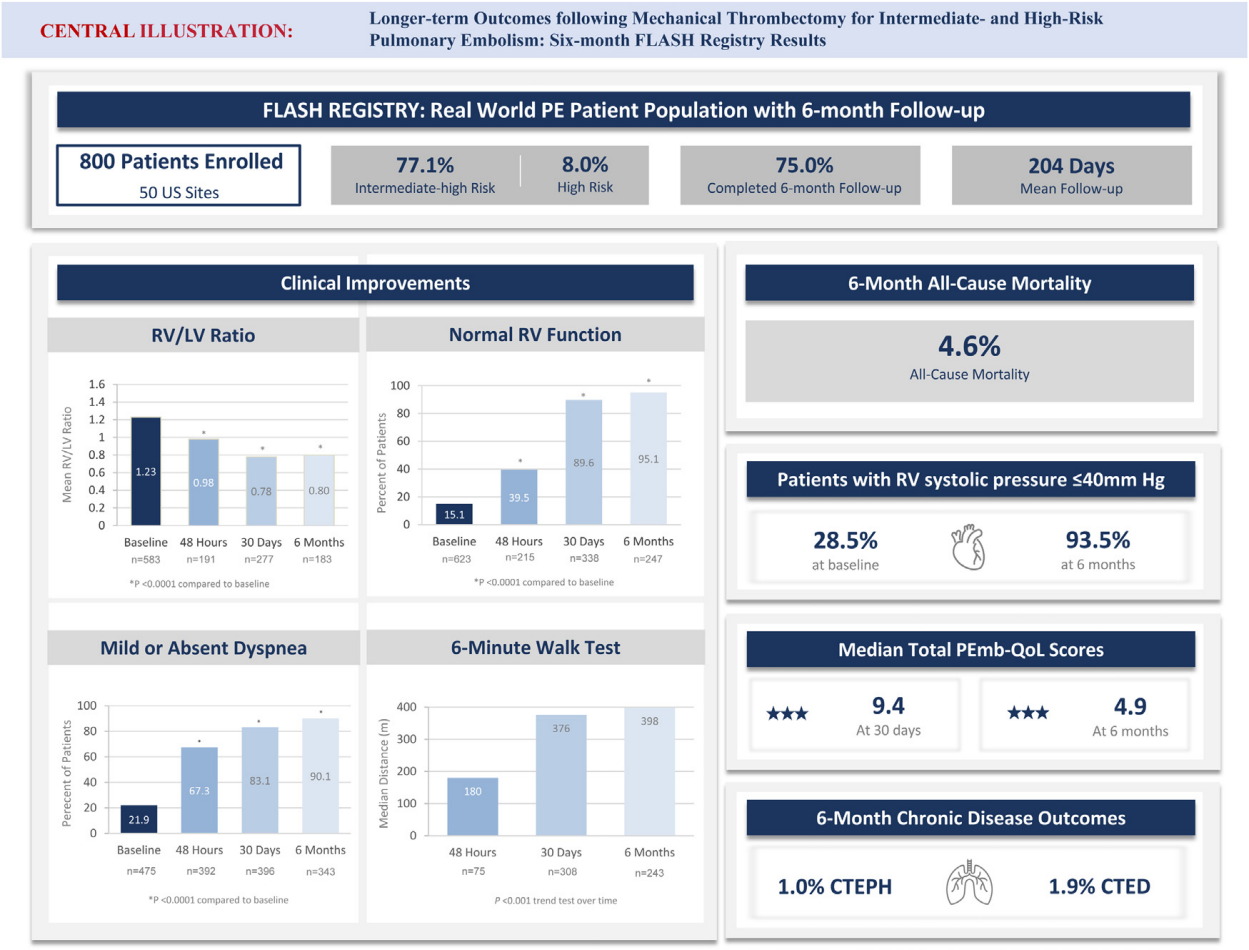
**Summary of patient enrollment, follow-up metrics, and 6-month clinical and functional outcomes for the fully enrolled US cohort of the FLASH registry in pulmonary embolism**. The FLASH registry enrolled 800 patients with acute pulmonary embolism across 50 US sites who were undergoing treatment with mechanical thrombectomy using the FlowTriever System and followed them through 6 months postthrombectomy. Follow-up completion and mean duration of follow-up are reported, along with 6-month all-cause mortality. Clinical and functional outcomes assessed from baseline through 6 months include echocardiographic measurements of RV size and function, dyspnea using the modified Medical Research Council score, exercise capacity using the 6-minute walk test, quality of life using the PEmb-QoL score, and prevalence of chronic disease outcomes including CTEPH or CTED. CTED, chronic thromboembolic disease; CTEPH, chronic thromboembolic pulmonary hypertension; FLASH, FlowTriever All-comer Registry for Patient Safety and Hemodynamics; PEmb-QoL, Pulmonary Embolism Quality of Life; RV, right ventricular.

## Discussion

The FLASH study is the largest prospective catheter-based interventional study of PE to evaluate short- and now longer-term outcomes among a geographically diverse and pragmatic population of US patients. There is an established body of evidence from FLASH as well as other single-center and multicenter studies reporting the acute hemodynamic improvements and favorable safety profile of FlowTriever mechanical thrombectomy for treatment of acute PE,^4^, ^5^, ^6^, ^7^, ^8^, ^9^, ^10^ but data describing longer-term clinical outcomes of this interventional treatment are limited by comparison. In addition to assessing acute benefits, the FLASH study also followed patients for 6 months postthrombectomy to provide longer-term follow-up data, addressing an important gap in the clinical evidence available for PE treatment with mechanical thrombectomy. In this report, patients treated with mechanical thrombectomy had improved echocardiographic parameters during 6-month follow-up. Perhaps more importantly to patients, the patient-reported outcomes including dyspnea score, QoL, and 6MWT distance all improved quickly after treatment, and these improvements were sustained throughout follow-up.

Echocardiographic measurements of RV parameters all showed significant improvements within 48 hours that continued out to 6 months following FlowTriever treatment, with 95.1% of patients having normal RV function at 6 months. Patients also experienced longer-term improvements in dyspnea, exercise capacity, and QoL over the follow-up period, including improvement in the median PEmb-QoL total score from 9.38 at 30 days to 4.85 at 6 months, improvement in 6MWT distances from 180 m at 48 hours to 398 m at 6 months, and significant reductions in both dyspnea and fatigue scores immediately following the 6MWT over time. Interestingly, most of the echocardiographic and functional improvements were achieved in the first 30 days with sustained benefit out to 6 months. These results support the hypothesis that early thrombus removal from pulmonary arteries reduces RV afterload, improves RV function, and helps patients experience early symptom relief that is maintained out to at least 6 months.

Patients are often hindered by significant and prolonged functional limitations after their initial PE, and it can take substantial time for patients to recover. Among patients treated with AC alone in the ELOPE study, approximately 50% of patients experienced significant exercise limitations after their initial PE, with symptoms and functional exercise capacity improving slowly over the first year.^1^ These results are supported by the FOCUS study, which found that almost 20% of patients met the diagnosis of PPEI over a 2-year follow-up, and these patients had reduced QoL and higher all-cause mortality compared with those without PPEI.^18^^,^^19^ The FOCUS study also noted a slow improvement of the PEmb-QoL scores over the course of the 2 years after a PE, with PPEI patients recording PEmb-QoL scores of 59.5, 46.9, and 23.3 at 3, 12, and 24 months, respectively. Patients without PPEI saw lower but also slowly improving scores of 20.7, 12.3, and 9.8 over the same time period.^18^^,^^19^ Patients with PE enrolled in the ELOPE study demonstrated a mean baseline PEmb-QoL score of 45.2, with total reductions of 11.0, 20.8, 25.4, and 32.1 points at 1, 3, 6, and 12 months, respectively.^1^^,^^20^ The 6MWT results from the ELOPE study demonstrated a similar trajectory of slow improvement in walk distances over the 1-year post-PE follow-up, with total increases of 20.7, 30.1, and 40.0 m above the 1-month distance at 3, 6, and 12 months.^20^ These data suggest that PPEI is common and associated with poor functional outcomes and decreased QoL and that some patients will never return to their pre-PE baseline functional capacity when treated with AC alone. The present study suggests that patients undergoing large-bore mechanical thrombectomy with the FlowTriever System have significant improvements in their functional capacity by 30 days, considerably earlier than those treated with conventional therapy. This rapid attainment of near-normal values can potentially translate into lower health care costs and a quicker return to normal daily activities for patients treated with mechanical thrombectomy. It may also establish the importance of intermediate follow-up timepoints, such as 30 days in future PE studies in addition to the standard longer-term timepoints to differentiate those treatments that may offer a more expedient response.

Long-term functional outcomes following catheter-based intervention have not been well studied, and a limited volume of data exists. A recent propensity-matched retrospective analysis matched patients receiving catheter-directed therapies (96.8% of patients received CDT and 3.2% received mechanical thrombectomy) to patients receiving medical management only and found a numerical but not statistically significant difference in mean 6MWT distances at 1 year (362 m for catheter-directed therapies and 231 m for medical management).^21^ Results from 1-year follow-up of the OPTALYSE study showed a baseline 6MWT distance of 327 to 352 m across the 4 study groups, with increased 6MWT distance of approximately 26.7 meters from 30 to 90 days, and again from 90 days to 1 year.^22^ A more recent study of 190 patients demonstrated that endovascular therapy (CDT or mechanical thrombectomy) led to significantly higher 6MWT distances at 3-6 months in intermediate–high and high-risk patients with PE than those receiving medical therapy only (342 m vs 272 m, respectively).^23^ The median 6MWT distance of 398.1 m at 6 months in this report represents an increase of 218 m from 48 hours to 6 months, exceeding the published results discussed above. The PEmb-QoL results in this report also compare favorably to literature values, which are sparse for catheter-directed therapy studies. Patients’ PEmb-QoL scores improved following CDT in the OPTALYSE study by a mean of −3.1 points between each follow-up timepoint out to 1 year, although specific values were not provided.^22^

For those patients who survive PE, many will face the long-term sequelae of RPVO, which is one of the strongest predictors of a poor long-term prognosis.^24^ The incidence of RPVO following thrombolytic or AC treatment is up to 60% of patients with PE^25^, ^26^, ^27^ and is associated with a higher risk of venous thromboembolism recurrence,^26^^,^^28^^,^^29^ persistent RV dysfunction,^27^ development of CTEPH,^26^^,^^29^^,^^30^ and mortality.^24^ Interventional treatments, including thrombectomy, that can rapidly extract thrombus may reduce the likelihood of RPVO and its consequences, including CTED and CTEPH. The FLASH study had a reported CTED prevalence of 1.9% and CTEPH prevalence of 1.0% at 6 months, lower than previously published CTEPH prevalence ranges of 2% to 4%,^31^ possibly owing to the removal of acute thrombi and prevention of RPVO. It is important to note that since much of the FLASH study occurred during the COVID-19 pandemic, some of the symptoms related to longer-term consequences of PE may overlap with those of COVID-19, potentially leading to overstated impact of PE on functional outcomes.

The FLASH registry has certain limitations. As with most registries, a detailed treatment protocol was not specified. As a result, procedural details and laboratory testing were performed per local clinical practice and not standardized, so procedural and outcome variability may be higher compared with an investigational trial with formally prescribed procedural requirements. The site-reported nature of CTED, CTEPH, and echocardiographic assessments and the lack of residual thrombus burden measurement postthrombectomy are also limitations. In addition, because of changes in protocol requirements for some outcome measurements over the course of the study, as well as an inability of many patients to have in-person follow-up visits during the COVID-19 pandemic, certain evaluations that could not be performed in a telehealth appointment, such as echocardiography and 6MWT, were not collected at all follow-up visits, resulting in a lower cohort population for these measurements. Finally, because FLASH is a single-arm registry, definitive comparisons to outcomes of other treatment options including other interventions or conservative medical management cannot be made. Randomized controlled trials (RCTs) are needed to directly compare such outcomes. The currently enrolling PEERLESS RCT will compare acute and intermediate-term (30-day) clinical outcomes in patients with PE treated with the FlowTriever System vs CDT. The HI-PEITHO RCT is also currently enrolling to evaluate the benefit of intervention with the EKOS CDT system (Boston Scientific) vs standard medical management in preventing acute adverse clinical outcomes. The STORM-PE RCT will evaluate improvements in the surrogate measure of RV/LV ratio in patients with PE treated with aspiration thrombectomy using the Indigo Aspiration System (Penumbra, Inc) vs standard medical management. Finally, the PE-TRACT RCT will evaluate improvements in longer-term clinical outcomes in patients with PE treated with intervention vs standard medical management.

## Conclusions

Among a large and geographically diverse cohort of patients with acute intermediate- and high-risk PE treated with the FlowTriever System, all-cause mortality and prevalence of CTED and CTEPH were low at 6-month follow-up. Furthermore, patient-reported health status was markedly improved by multiple measures suggesting durable clinical benefits that compare favorably against other studies using similar measures. These data suggest that rapid extraction of thrombus may prevent or reduce long-term sequelae in patients with PE, informing the hypothesis for future studies directly comparing the clinical outcomes of prompt mechanical thrombectomy relative to conservative therapy for PE management.
