# Supplementary material for: Longer-Term Outcomes Following Mechanical Thrombectomy for Intermediate- and High-Risk Pulmonary Embolism: 6-Month FLASH Registry Results
Source: J Soc Cardiovasc Angiogr Interv. 2023 May 19;2(4):101000. doi: 10.1016/j.jscai.2023.101000 (PMC11307656; doi:10.1016/j.jscai.2023.101000)
Supplement: Supplemental Table S1-S3 [file mmc1.docx]

**SUPPLEMENTAL TABLE 1: Listing of Serious Adverse Events**

| **Serious Adverse Events** | **≤48 hours**  n=37 | **>48 hours to**  **30 days**  n=26 | **>30 days**  n=40 | **Total**  n=103 | **Relationship to device** |
| --- | --- | --- | --- | --- | --- |
| Acute pulmonary edema | 0 | 1 | 0 | 1 | unrelated |
| Acute respiratory failure | 2 | 0 | 1 | 3 | unrelated |
| Anemia | 4 | 4 | 0 | 8 | unrelated |
| Arterial repair | 0 | 1 | 0 | 1 | unrelated |
| Atrial fibrillation | 1 | 0 | 0 | 1 | unrelated |
| Atrial thrombosis | 1 | 0 | 0 | 1 | unrelated |
| Blood loss anemia | 1 | 0 | 0 | 1 | unrelated |
| Bradycardia | 1 | 0 | 1 | 2 | unrelated |
| Bundle branch block left | 1 | 0 | 0 | 1 | unrelated |
| Cardiac arrest | 0 | 0 | 5 | 5 | unrelated |
| Cardiac failure | 0 | 1 | 0 | 1 | unrelated |
| Cardiac procedure complication | 1 | 0 | 0 | 1 | unrelated |
| Cardiac valve rupture | 0 | 0 | 1 | 1 | unknown^a^ |
| Cardio-respiratory arrest | 1 | 0 | 1 | 2 | unrelated |
| Cerebrovascular accident | 1 | 0 | 0 | 1 | unrelated |
| Chest discomfort | 1 | 0 | 0 | 1 | unrelated |
| Cholangiocarcinoma | 0 | 0 | 1 | 1 | unrelated |
| Colon cancer metastatic / Colorectal cancer stage IV | 0 | 0 | 2 | 2 | unrelated |
| Death^b^ | 0 | 2 | 1 | 3 | unrelated |
| Deep vein thrombosis | 2 | 2 | 3 | 7 | unrelated |
| Groin abscess | 0 | 0 | 1 | 1 | unrelated |
| Hemoglobin decreased | 1 | 0 | 0 | 1 | unrelated |
| Hemoptysis | 0 | 1 | 0 | 1 | unrelated |
| Hemorrhage | 1 | 0 | 0 | 1 | unrelated |
| Hemorrhagic shock | 0 | 1 | 0 | 1 | unrelated |
| Heparin-induced thrombocytopenia | 0 | 2 | 0 | 2 | unrelated |
| Hypotension | 1 | 0 | 0 | 1 | unrelated |
| Hypovolemic shock | 1 | 0 | 0 | 1 | unrelated |
| Hypoxia | 0 | 0 | 1 | 1 | unrelated |
| Intestinal perforation | 0 | 1 | 0 | 1 | unrelated |
| Ischemic stroke | 0 | 1 | 0 | 1 | unrelated |
| Lymphoproliferative disorder | 0 | 0 | 1 | 1 | unrelated |
| Mental status changes | 0 | 1 | 0 | 1 | unknown^a^ |
| Metabolic encephalopathy | 0 | 0 | 1 | 1 | unrelated |
| Overdose | 0 | 0 | 1 | 1 | unrelated |
| Pancreatic carcinoma, metastatic | 0 | 0 | 1 | 1 | unrelated |
| Peripheral ischemia | 1 | 0 | 0 | 1 | unrelated |
| Plasma cell myeloma | 0 | 0 | 2 | 2 | unrelated |
| Pneumonia | 0 | 0 | 1 | 1 | unrelated |
| Prostate cancer, metastatic | 0 | 0 | 1 | 1 | unrelated |
| Pulmonary embolism | 2 | 2 | 6 | 10 | unrelated |
| Pulmonary embolism, post-procedural | 0 | 0 | 1 | 1 | unrelated |
| Respiratory failure | 0 | 0 | 1 | 1 | unrelated |
| Retroperitoneal hematoma/hemorrhage | 2 | 1 | 0 | 3 | unrelated |
| Sepsis/septic shock | 0 | 2 | 0 | 2 | unrelated |
| Shock | 1 | 0 | 0 | 1 | unrelated |
| Squamous cell carcinoma, metastatic | 0 | 0 | 1 | 1 | unrelated |
| Subdural hematoma | 0 | 0 | 2 | 2 | unrelated |
| Tachycardia | 1 | 0 | 0 | 1 | unrelated |
| Uterine cancer | 0 | 0 | 1 | 1 | unrelated |
| Vaginal hemorrhage | 0 | 1 | 0 | 1 | unrelated |
| Vascular access site complication | 0 | 1 | 0 | 1 | unrelated |
| Vascular access site hematoma | 4 | 1 | 0 | 5 | unrelated |
| Vascular access site hemorrhage | 3 | 0 | 0 | 3 | unrelated |
| Vascular pseudoaneurysm | 1 | 0 | 2 | 3 | unrelated |
| Ventricular tachycardia | 1 | 0 | 0 | 1 | unrelated |
| SAE terms follow Medical Dictionary for Regulatory Activities (MedDRA) terminology.  ^a^Determined by independent medical monitor to be of unknown device-relatedness.  ^b^Three (3) deaths were classified as General Disorders per MedDRA. The remaining 26 deaths in this study were outcomes of other diseases or conditions listed within this table. | | | | | |

**SUPPLEMENTAL TABLE 2: Anticoagulant Use at 30-day and 6-month Visit**

| **Anticoagulant Type** | **30d Visit**  n=661 | **6mo Visit**  n=546 |
| --- | --- | --- |
| Unfractionated heparin | 4 (0.6%) | 3 (0.5%) |
| Low molecular weight heparin | 22 (3.3%) | 6 (1.1%) |
| Vitamin K antagonist | 64 (9.7%) | 52 (9.5%) |
| NOAC/DOAC | 565 (85.5%) | 476 (87.2%) |
| Other | 17 (2.6%) | 15 (2.7%) |
| Some patients were on more than one type of anticoagulant. Percentages may total to more than 100%. | | |

**SUPPLEMENTAL TABLE 3: Principal Investigators (PI) and Sites**

| **Principal Investigators** | **Site** |
| --- | --- |
| Toma, Catalin (Global FLASH PI) | University of Pittsburgh Medical Center (Pittsburgh, PA) |
| Jaber, Wissam | Emory University (Atlanta, GA) |
| Weinberg, Mitchell | Northwell Health (Staten Island, NY) |
| Bunte, Matthew; Cho, Kenneth^a^ | Saint Luke's MidAmerica Heart Institute (Kansas City, MO) |
| Stegman, Brian | CentraCare Heart & Vascular Center (St. Cloud, MN) |
| Khandhar, Sameer | University of Pennsylvania (Philadelphia, PA) |
| Gondi, Sreedevi | Baptist Health Louisville (Louisville, KY) |
| Chambers, Jeffrey | Metropolitan Heart & Vascular Institute (Minneapolis, MN) |
| Amin, Rohit | Ascension Sacred Heart Hospital (Pensacola, FL) |
| Leung, Daniel | Christiana Care Health Services (Newark, DE) |
| Pollak, Jeffrey | Yale University (New Haven, CT) |
| Kado, Herman | Ascension Providence Hospital (Southfield, MI) |
| Sarosi, Michael | St. Joseph Mercy Hospital (Ann Arbor, MI) |
| Brown, Michael | Missouri Cardiovascular Specialists (Columbia, MO) |
| Castle, Jordan | Inland Imaging, Providence Sacred Heart (Spokane, WA) |
| Bhat, Ambarish | University of Missouri, Columbia (Columbia, MO) |
| Savin, Michael | Oakland University William Beaumont School of Medicine (Royal Oak, MI) |
| Siskin, Gary | Albany Medical Center (Albany, NY) |
| Beam, Daren; Stewart, Lauren Kathryn | Indiana University School of Medicine (Indianapolis, IN) |
| Brancheau, Daniel | Ascension Genesys Hospital (Grand Blanc, MI) |
| Ahmed, Mustafa | UAB Division of Cardiovascular Disease (Birmingham, AL) |
| Rosenberg, Michael; Fanola, Christina | University of Minnesota (Minneapolis, MN) |
| Elmasri, Fakhir | Lakeland Vascular Institute (Lakeland, FL) |
| Zlotnick, David | SUNY, The University at Buffalo (Buffalo, NY) |
| Bisharat, Mohannad | Memorial Hospital Jacksonville (Jacksonville, FL) |
| DuCoffe, Aaron; ^a^Butros, Paul | Inova Health System (Fairfax, VA) |
| Koenig, Gerald | Henry Ford Health System (Detroit, MI) |
| Zybulewski, Adam; Olivieri, Brandon^a^, Beasley, Robert^a^ | Mount Sinai Medical Center of Florida (Miami, FL) |
| Angel, Wesley; Roberts, Jon | Methodist Healthcare Foundation (Germantown, TN) |
| Kerrigan, Jimmy | Ascension St. Thomas West (Nashville, TN) |
| Li, Jun | University Hospitals Cleveland Medical Center (Cleveland, OH) |
| Schimmel, Daniel | Northwestern University (Chicago, IL) |
| Balderman, Joshua | Pima Heart and Vascular (Tucson, AZ) |
| Patton, Marquand | Palmetto General Hospital (Hialeah, FL) |
| Walker, Chris | University of Tennessee Medical Center Knoxville (Knoxville, TN) |
| Gonsalves, Carin | Thomas Jefferson University (Philadelphia, PA) |
| Kasmikha, Zaid; Qaqi, Osama^a^ | Ascension Providence Rochester Hospital (Rochester, MI) |
| Veerina, Kalyan | Opelousas General Hospital (Opelousas, LA) |
| Dexter, David | Sentara Vascular Specialists (Norfolk, VA) |
| Lookstein, Robert | Mount Sinai Medical Center (New York, NY) |
| Lasic, Zoran | Jamaica Hospital/Lenox Hill (New York, NY) |
| Mahjoobi, Maziar | CardioVoyage Hospital (Denison, TX) |
| Paul, Jonathan | University of Chicago (Chicago, IL) |
| Tumuluri, Ramagopal | Aurora St. Luke’s Medical Center (Milwaukee, WI) |
| Horowitz, James | NYU Langone Medical Center (New York, NY) |
| Lee, Justin | Sarasota Memorial (Sarasota, FL) |
| Leonardi, Robert | Lexington Medical Center (Lexington, SC) |
| Patel, Mitul | Valley Health (Ridgewood, NJ) |
| Rothschild, Daniel | Norton Healthcare (Louisville, KY) |
| Tabriz, David | Rush University Medical Center (Chicago, IL) |
| ^a^Past Principal Investigators |  |
